# *O*-4-Chloro­benzoyl diphenyl­seleno­phosphinate

**DOI:** 10.1107/S1600536809009313

**Published:** 2009-03-25

**Authors:** Grzegorz Cholewinski, Jaroslaw Chojnacki, Jerzy Pikies, Janusz Rachon

**Affiliations:** a Chemical Faculty, Gdansk University of Technology, Narutowicza 11/12, Gdansk PL-80233, Poland

## Abstract

The title compound, C_19_H_14_ClO_2_PSe, was obtained in the reaction of the diphenyl­monoseleno­phosphinic acid ammonium salt with 4-chloro­benzoyl chloride. The dihedral angle between the P-bonded aromatic rings is 72.64 (14)°. Packing of the mol­ecules in the crystal is reinforced by π–π stacking inter­actions between two inversion-related 4-chloro­benzene rings [centroid-centroid separation = 4.189 (2) Å] and a C—H⋯O interaction also occurs.

## Related literature

Syntheses of *O*-acyl monoseleno­phosphates have already been described by Rachon *et al.* (2005 ▶); Mielniczak & Łopusinski (2001 ▶). For a related *O*-acyl derivative, see Cholewinski *et al.* (2009 ▶). For related *O*-alkyl or *O*-aryl derivatives, see: Lepicard *et al.* (1969 ▶); Balakrishna *et al.* (2002 ▶, 2005 ▶); Mague *et al.* (2007 ▶). 
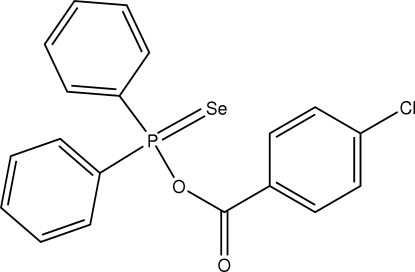


## Experimental

### 

#### Crystal data

C_19_H_14_ClO_2_PSe
*M* *_r_* = 419.68
Monoclinic, 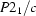

*a* = 9.3390 (5) Å
*b* = 9.7132 (5) Å
*c* = 19.1353 (15) Å
β = 97.059 (6)°
*V* = 1722.64 (19) Å^3^
*Z* = 4
Mo *K*α radiation
μ = 2.44 mm^−1^
*T* = 120 K
0.46 × 0.33 × 0.26 mm

#### Data collection

Oxford Diffraction KM-4-CCD diffractometer
Absorption correction: multi-scan (*CrysAlis RED*; Oxford Diffraction, 2008 ▶) *T*_min_ = 0.325, *T*_max_ = 0.53
14128 measured reflections
4158 independent reflections
3376 reflections with *I* > 2σ(*I*)
*R*_int_ = 0.045

#### Refinement

*R*[*F* ^2^ > 2σ(*F* ^2^)] = 0.054
*wR*(*F* ^2^) = 0.162
*S* = 1.16
4158 reflections
217 parameters
H-atom parameters constrained
Δρ_max_ = 1.82 e Å^−3^
Δρ_min_ = −0.56 e Å^−3^

### 

Data collection: *CrysAlis CCD* (Oxford Diffraction, 2008 ▶); cell refinement: *CrysAlis RED* (Oxford Diffraction, 2008 ▶); data reduction: *CrysAlis RED*; program(s) used to solve structure: *SHELXS97* (Sheldrick, 2008 ▶); program(s) used to refine structure: *SHELXL97* (Sheldrick, 2008 ▶); molecular graphics: *ORTEP-3 for Windows* (Farrugia, 1997 ▶); software used to prepare material for publication: *WinGX* (Farrugia, 1999 ▶) and *PLATON* (Spek, 2009 ▶).

## Supplementary Material

Crystal structure: contains datablocks global, I. DOI: 10.1107/S1600536809009313/ez2164sup1.cif

Structure factors: contains datablocks I. DOI: 10.1107/S1600536809009313/ez2164Isup2.hkl

Additional supplementary materials:  crystallographic information; 3D view; checkCIF report

## Figures and Tables

**Table 1:** Hydrogen-bond geometry (Å, °)

*D*—H⋯*A*	*D*—H	H⋯*A*	*D*⋯*A*	*D*—H⋯*A*
C16—H16⋯O2^i^	0.95	2.59	3.359 (6)	138

Symmetry code: (i) 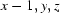.

**Fig. 1.**
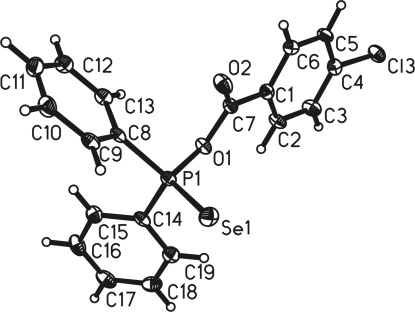
View of the title compound showing the atom-numbering scheme (50% probability displacement ellipsoids).

## supplementary crystallographic information

### Comment

*O*-acyl monoselenophosphates were studied as part of a search for potential selenoacylating agents. *O*-4-chlorobenzoyl-diphenylmonoselenophosphinate was obtained in the reaction of diphenylmonoselenophosphinic acid ammonium salt with 4-chlorobenzoyl chloride (Rachon *et al.* 2005).

The title compound, C_19_H_14_ClO_2_PSe, together with *O*-pivaloyl- diphenylselenophosphinate, reported in our preceding paper (Cholewinski *et al.*, 2009) are the first reported X-ray diffraction structures of *O*-acyl derivatives of diphenylmonoselenophosphinic acid. Several other monoselenophosphinates, such as *O*-alkyl esters have been characterized by X-ray diffraction (Lepicard *et al.*, 1969; Balakrishna *et al.*, 2005; Balakrishna *et al.*, 2002; Mague *et al.*, 2007).

The P–Se bond length is in the usual range, while the P–O bond is longer than in *O*-aryl or *O*-alkyl derivatives, but very close to the value for the other *O*-acyl derivative, reported in our preceding paper (Cholewinski *et al.*, 2009). Unlike the pivaloyl analogue, no phenyl ring is close to being synplanar with the Se–P–C plane. The smallest Se–P–C–C torsion angle is relatively large at 25.7 (4)°. Noteworthy is that the carboxyl group is twisted in relation to the adjacent phenyl ring, forming a dihedral angle between the C1–C6 phenyl ring and the plane defined by O1/C7/O2 of 11.6 (7)°.

Packing of *O*-4-chlorobenzoyl-diphenylmonoselenophosphinate molecules in the crystal is reinforced by π - π stacking interactions between two adjacent 4-chlorobenzene rings residing close to a symmetry centre. The centroid to centroid distance is 4.189 (2) Å, the dihedral angle between the planes is α=0.0° and the angle between the vector span on the centroids and the vector normal to the ring is β=35.08°. No classical hydrogen bonds are present, however a weak C–H···O interaction link molecules into chains along the a-axis.

### Experimental

*O*-4-chlorobenzoyl-diphenylmonoselenophosphinate was obtained in the reaction of diphenylmonoselenophosphinic acid ammonium salt (Mielniczak *et al.*, 2001) with 4-chlorobenzoyl chloride in 46% yield according to Rachon *et al.* (2005). The compound which was encoded in this paper as 2t, melts at 105-107 °C. Relevant ^1^H, ^13^C, ^31^P NMR, MS and IR spectra were recorded and are consistent with the formula anticipated - see the supporting information for the article cited.

### Refinement

Hydrogen atoms were placed in calculated positions and refined using a standard riding model. C–H bond lengths were set to 0.95Å and *U**_iso_*(H) were set to 1.2*U**_eq_*(C) for aromatic C–H groups, respectively. The residual electron-density peak is 0.95Å from Se1 and the deepest electron-density hole is 1.49Å from Se1.

### Crystal data

**Table:**

C_19_H_14_ClO_2_PSe	*F*(000) = 840
*M**_r_* = 419.68	*D*_x_ = 1.618 Mg m^−^^3^
Monoclinic, *P*2_1_/*c*	Melting point: 379(2) K
Hall symbol: -P 2ybc	Mo *K*α radiation, λ = 0.71073 Å
*a* = 9.3390 (5) Å	Cell parameters from 9084 reflections
*b* = 9.7132 (5) Å	θ = 2.1–32.4°
*c* = 19.1353 (15) Å	µ = 2.44 mm^−^^1^
β = 97.059 (6)°	*T* = 120 K
*V* = 1722.64 (19) Å^3^	Prism, colourless
*Z* = 4	0.46 × 0.33 × 0.26 mm

### Data collection

**Table:**

Oxford Diffraction KM-4-CCD diffractometer	4158 independent reflections
Radiation source: enhance	3376 reflections with *I* > 2σ(*I*)
graphite	*R*_int_ = 0.045
ω scans (0.75° width)	θ_max_ = 28°, θ_min_ = 2.1°
Absorption correction: multi-scan (*CrysAlis RED*; Oxford Diffraction, 2008)	*h* = −12→12
*T*_min_ = 0.325, *T*_max_ = 0.53	*k* = −12→12
14128 measured reflections	*l* = −21→25

### Refinement

**Table:**

Refinement on *F*^2^	Primary atom site location: structure-invariant direct methods
Least-squares matrix: full	Secondary atom site location: difference Fourier map
*R*[*F*^2^ > 2σ(*F*^2^)] = 0.054	Hydrogen site location: inferred from neighbouring sites
*wR*(*F*^2^) = 0.162	H-atom parameters constrained
*S* = 1.16	*w* = 1/[σ^2^(*F*_o_^2^) + (0.0811*P*)^2^ + 5.5587*P*] where *P* = (*F*_o_^2^ + 2*F*_c_^2^)/3
4158 reflections	(Δ/σ)_max_ = 0.001
217 parameters	Δρ_max_ = 1.82 e Å^−^^3^
0 restraints	Δρ_min_ = −0.56 e Å^−^^3^

### Special details

**Table:**

Geometry. All e.s.d.'s (except the e.s.d. in the dihedral angle between two l.s. planes) are estimated using the full covariance matrix. The cell e.s.d.'s are taken into account individually in the estimation of e.s.d.'s in distances, angles and torsion angles; correlations between e.s.d.'s in cell parameters are only used when they are defined by crystal symmetry. An approximate (isotropic) treatment of cell e.s.d.'s is used for estimating e.s.d.'s involving l.s. planes.

### Fractional atomic coordinates and isotropic or equivalent isotropic displacement parameters (Å^2^)

**Table:**

	*x*	*y*	*z*	*U*_iso_*/*U*_eq_	
Se1	0.28670 (5)	1.03290 (5)	0.35818 (2)	0.02233 (16)	
Cl3	0.57203 (12)	0.18536 (11)	0.48614 (6)	0.0244 (2)	
P1	0.15102 (11)	0.87791 (11)	0.31430 (5)	0.0146 (2)	
O1	0.2030 (3)	0.7199 (3)	0.33713 (15)	0.0179 (6)	
O2	0.3944 (3)	0.7246 (4)	0.27566 (17)	0.0242 (7)	
C1	0.3808 (4)	0.5449 (4)	0.3593 (2)	0.0167 (8)	
C2	0.3182 (5)	0.5004 (5)	0.4180 (2)	0.0206 (9)	
H2	0.2362	0.5467	0.4314	0.025*	
C3	0.3764 (5)	0.3881 (5)	0.4567 (2)	0.0232 (9)	
H3	0.3361	0.3584	0.4974	0.028*	
C4	0.4933 (5)	0.3202 (4)	0.4353 (2)	0.0186 (8)	
C5	0.5533 (5)	0.3590 (5)	0.3756 (2)	0.0197 (8)	
H5	0.631	0.3083	0.3606	0.024*	
C6	0.4977 (5)	0.4734 (5)	0.3380 (2)	0.0196 (8)	
H6	0.5394	0.5031	0.2977	0.023*	
C7	0.3321 (4)	0.6701 (5)	0.3192 (2)	0.0177 (8)	
C8	0.1201 (4)	0.8755 (4)	0.2195 (2)	0.0136 (7)	
C9	0.1340 (5)	0.9975 (4)	0.1825 (2)	0.0192 (8)	
H9	0.1618	1.0799	0.2072	0.023*	
C10	0.1069 (5)	0.9982 (5)	0.1093 (2)	0.0239 (9)	
H10	0.1154	1.0814	0.0841	0.029*	
C11	0.0675 (5)	0.8778 (5)	0.0733 (2)	0.0235 (9)	
H11	0.0511	0.878	0.0233	0.028*	
C12	0.0521 (5)	0.7567 (5)	0.1102 (2)	0.0214 (9)	
H12	0.0237	0.6745	0.0852	0.026*	
C13	0.0776 (4)	0.7549 (4)	0.1828 (2)	0.0186 (8)	
H13	0.0663	0.6718	0.2078	0.022*	
C14	−0.0242 (4)	0.8745 (4)	0.3454 (2)	0.0156 (8)	
C15	−0.1503 (5)	0.8706 (5)	0.2982 (2)	0.0206 (9)	
H15	−0.1454	0.8667	0.2489	0.025*	
C16	−0.2835 (5)	0.8724 (5)	0.3239 (3)	0.0261 (10)	
H16	−0.37	0.8687	0.2922	0.031*	
C17	−0.2900 (5)	0.8795 (5)	0.3957 (3)	0.0259 (10)	
H17	−0.3812	0.882	0.4128	0.031*	
C18	−0.1647 (5)	0.8830 (5)	0.4427 (2)	0.0236 (9)	
H18	−0.17	0.8872	0.4919	0.028*	
C19	−0.0315 (5)	0.8803 (5)	0.4177 (2)	0.0204 (8)	
H19	0.0547	0.8824	0.4497	0.024*	

### Atomic displacement parameters (Å^2^)

**Table:**

	*U*^11^	*U*^22^	*U*^33^	*U*^12^	*U*^13^	*U*^23^
Se1	0.0197 (2)	0.0211 (3)	0.0260 (3)	−0.00604 (17)	0.00179 (16)	−0.00448 (17)
Cl3	0.0239 (5)	0.0179 (5)	0.0317 (6)	0.0050 (4)	0.0049 (4)	0.0057 (4)
P1	0.0138 (5)	0.0119 (5)	0.0183 (5)	−0.0001 (4)	0.0032 (4)	0.0002 (4)
O1	0.0148 (13)	0.0164 (15)	0.0231 (14)	0.0027 (11)	0.0044 (11)	0.0033 (11)
O2	0.0193 (15)	0.0260 (17)	0.0286 (16)	0.0038 (13)	0.0079 (12)	0.0092 (13)
C1	0.0147 (18)	0.015 (2)	0.0198 (19)	−0.0007 (15)	0.0009 (15)	−0.0001 (15)
C2	0.0164 (19)	0.020 (2)	0.027 (2)	0.0034 (16)	0.0084 (16)	0.0015 (17)
C3	0.023 (2)	0.022 (2)	0.027 (2)	−0.0003 (18)	0.0119 (17)	0.0052 (18)
C4	0.0179 (19)	0.0130 (19)	0.024 (2)	−0.0018 (16)	−0.0004 (15)	0.0008 (16)
C5	0.0167 (19)	0.019 (2)	0.024 (2)	0.0048 (16)	0.0042 (15)	−0.0031 (16)
C6	0.019 (2)	0.022 (2)	0.0178 (19)	0.0028 (17)	0.0034 (15)	−0.0014 (16)
C7	0.0133 (18)	0.020 (2)	0.0196 (19)	0.0015 (16)	0.0013 (14)	−0.0012 (16)
C8	0.0096 (16)	0.0099 (18)	0.0217 (19)	0.0005 (14)	0.0036 (14)	0.0013 (15)
C9	0.020 (2)	0.0115 (19)	0.026 (2)	0.0035 (16)	0.0041 (16)	0.0015 (15)
C10	0.026 (2)	0.020 (2)	0.025 (2)	0.0035 (18)	0.0029 (17)	0.0077 (17)
C11	0.023 (2)	0.027 (2)	0.020 (2)	0.0013 (19)	0.0012 (16)	0.0019 (18)
C12	0.021 (2)	0.017 (2)	0.026 (2)	−0.0016 (17)	0.0017 (16)	−0.0032 (17)
C13	0.0181 (19)	0.0122 (19)	0.026 (2)	−0.0018 (16)	0.0039 (15)	−0.0005 (16)
C14	0.0144 (18)	0.0081 (17)	0.025 (2)	0.0001 (14)	0.0047 (15)	0.0018 (15)
C15	0.018 (2)	0.021 (2)	0.023 (2)	0.0001 (17)	0.0035 (16)	0.0026 (17)
C16	0.0127 (19)	0.030 (3)	0.036 (2)	0.0012 (18)	0.0030 (17)	0.007 (2)
C17	0.020 (2)	0.021 (2)	0.039 (3)	0.0026 (18)	0.0132 (18)	0.0050 (19)
C18	0.029 (2)	0.020 (2)	0.024 (2)	−0.0018 (18)	0.0112 (17)	−0.0012 (17)
C19	0.020 (2)	0.017 (2)	0.024 (2)	−0.0010 (17)	0.0035 (16)	−0.0006 (16)

### Geometric parameters (Å, °)

**Table:**

Se1—P1	2.0774 (11)	C9—C10	1.392 (6)
Cl3—C4	1.740 (4)	C9—H9	0.95
P1—O1	1.653 (3)	C10—C11	1.384 (7)
P1—C8	1.802 (4)	C10—H10	0.95
P1—C14	1.809 (4)	C11—C12	1.388 (6)
O1—C7	1.380 (5)	C11—H11	0.95
O2—C7	1.197 (5)	C12—C13	1.382 (6)
C1—C6	1.396 (6)	C12—H12	0.95
C1—C2	1.397 (6)	C13—H13	0.95
C1—C7	1.479 (6)	C14—C15	1.394 (6)
C2—C3	1.392 (6)	C14—C19	1.394 (6)
C2—H2	0.95	C15—C16	1.393 (6)
C3—C4	1.380 (6)	C15—H15	0.95
C3—H3	0.95	C16—C17	1.383 (7)
C4—C5	1.385 (6)	C16—H16	0.95
C5—C6	1.389 (6)	C17—C18	1.387 (7)
C5—H5	0.95	C17—H17	0.95
C6—H6	0.95	C18—C19	1.387 (6)
C8—C9	1.395 (6)	C18—H18	0.95
C8—C13	1.398 (6)	C19—H19	0.95
			
O1—P1—C8	105.05 (17)	C10—C9—H9	120
O1—P1—C14	98.28 (17)	C8—C9—H9	120
C8—P1—C14	106.94 (18)	C11—C10—C9	120.0 (4)
O1—P1—Se1	114.93 (11)	C11—C10—H10	120
C8—P1—Se1	115.52 (13)	C9—C10—H10	120
C14—P1—Se1	114.32 (14)	C10—C11—C12	120.1 (4)
C7—O1—P1	119.8 (3)	C10—C11—H11	119.9
C6—C1—C2	120.0 (4)	C12—C11—H11	119.9
C6—C1—C7	117.4 (4)	C13—C12—C11	120.4 (4)
C2—C1—C7	122.6 (4)	C13—C12—H12	119.8
C3—C2—C1	119.8 (4)	C11—C12—H12	119.8
C3—C2—H2	120.1	C12—C13—C8	119.9 (4)
C1—C2—H2	120.1	C12—C13—H13	120.1
C4—C3—C2	119.2 (4)	C8—C13—H13	120.1
C4—C3—H3	120.4	C15—C14—C19	120.2 (4)
C2—C3—H3	120.4	C15—C14—P1	120.9 (3)
C3—C4—C5	122.0 (4)	C19—C14—P1	118.8 (3)
C3—C4—Cl3	119.3 (3)	C16—C15—C14	119.4 (4)
C5—C4—Cl3	118.7 (3)	C16—C15—H15	120.3
C4—C5—C6	118.8 (4)	C14—C15—H15	120.3
C4—C5—H5	120.6	C17—C16—C15	120.1 (4)
C6—C5—H5	120.6	C17—C16—H16	119.9
C5—C6—C1	120.2 (4)	C15—C16—H16	119.9
C5—C6—H6	119.9	C16—C17—C18	120.6 (4)
C1—C6—H6	119.9	C16—C17—H17	119.7
O2—C7—O1	122.2 (4)	C18—C17—H17	119.7
O2—C7—C1	125.4 (4)	C19—C18—C17	119.8 (4)
O1—C7—C1	112.4 (3)	C19—C18—H18	120.1
C9—C8—C13	119.7 (4)	C17—C18—H18	120.1
C9—C8—P1	119.1 (3)	C18—C19—C14	119.9 (4)
C13—C8—P1	121.1 (3)	C18—C19—H19	120
C10—C9—C8	119.9 (4)	C14—C19—H19	120
			
C8—P1—O1—C7	65.5 (3)	Se1—P1—C8—C13	156.6 (3)
C14—P1—O1—C7	175.7 (3)	C13—C8—C9—C10	−0.7 (6)
Se1—P1—O1—C7	−62.6 (3)	P1—C8—C9—C10	−178.4 (3)
C6—C1—C2—C3	2.5 (7)	C8—C9—C10—C11	−0.5 (7)
C7—C1—C2—C3	−175.0 (4)	C9—C10—C11—C12	1.2 (7)
C1—C2—C3—C4	−1.5 (7)	C10—C11—C12—C13	−0.8 (7)
C2—C3—C4—C5	−1.3 (7)	C11—C12—C13—C8	−0.4 (6)
C2—C3—C4—Cl3	176.7 (4)	C9—C8—C13—C12	1.1 (6)
C3—C4—C5—C6	2.9 (7)	P1—C8—C13—C12	178.8 (3)
Cl3—C4—C5—C6	−175.0 (3)	O1—P1—C14—C15	−108.5 (4)
C4—C5—C6—C1	−1.9 (7)	C8—P1—C14—C15	0.1 (4)
C2—C1—C6—C5	−0.8 (7)	Se1—P1—C14—C15	129.3 (3)
C7—C1—C6—C5	176.8 (4)	O1—P1—C14—C19	73.5 (4)
P1—O1—C7—O2	−15.8 (6)	C8—P1—C14—C19	−177.9 (3)
P1—O1—C7—C1	164.5 (3)	Se1—P1—C14—C19	−48.7 (4)
C6—C1—C7—O2	−9.5 (7)	C19—C14—C15—C16	0.0 (7)
C2—C1—C7—O2	168.1 (5)	P1—C14—C15—C16	−178.0 (4)
C6—C1—C7—O1	170.3 (4)	C14—C15—C16—C17	0.6 (7)
C2—C1—C7—O1	−12.2 (6)	C15—C16—C17—C18	−0.9 (8)
O1—P1—C8—C9	−153.4 (3)	C16—C17—C18—C19	0.5 (7)
C14—P1—C8—C9	102.8 (3)	C17—C18—C19—C14	0.1 (7)
Se1—P1—C8—C9	−25.7 (4)	C15—C14—C19—C18	−0.3 (7)
O1—P1—C8—C13	28.9 (4)	P1—C14—C19—C18	177.6 (4)
C14—P1—C8—C13	−74.9 (4)		

### Hydrogen-bond geometry (Å, °)

**Table:**

*D*—H···*A*	*D*—H	H···*A*	*D*···*A*	*D*—H···*A*
C16—H16···O2^i^	0.95	2.59	3.359 (6)	138

Symmetry codes: (i) *x*−1, *y*, *z*.
